# *“These are people just like us who can work”*: Overcoming clinical resistance and shifting views in the implementation of Individual Placement and Support (IPS)

**DOI:** 10.1007/s10488-022-01204-3

**Published:** 2022-07-05

**Authors:** Danika Sharek, Niamh Lally, Ciara Brennan, Agnes Higgins

**Affiliations:** 1 Dublin, Ireland; 2 Centre for Social Innovation, Trinity Business School, Trinity College, Dublin, Ireland; 3 School of Nursing and Midwifery, Trinity College Dublin, Dublin, Ireland

**Keywords:** Individual Placement and Support (IPS), Mental health service reform, Multi-disciplinary teams (MDT), Recovery, Republic of Ireland

## Abstract

**Purpose:**

Individual Placement and Support (IPS) is a recovery-based approach to support people with mental health difficulties back into employment. Embedding of IPS within the mental health Multi-Disciplinary Team (MDT) is a key component of IPS fidelity; however, few studies have examined how those involved with IPS implementation navigate this process. This article explores how IPS Employment Specialists (ES) and Occupational Therapist (OT) Managers integrated and embedded IPS within traditionally-oriented MDTs as part of a national reform program in the Republic of Ireland.

**Methods:**

The study design was qualitative, descriptive with data collected through three focus groups with 17 IPS ESs and 11 OT Managers. Data were analyzed using thematic synthesis.

**Results:**

Three key themes emerged from analysis. The first characterizes the context into which IPS landed, described as one marked by clinical resistance, doubt, and fear of risk. The second explores the strategies and factors that helped with the introduction of IPS into Irish mental health services. These included strategies, such as providing education and information about IPS and reassuring the MDT about IPS governance and IPS ES’ competencies. The evidenced-based nature of IPS and its attached accountabilities through IPS fidelity measures were perceived to be an important factor in its acceptance. The final theme encapsulates perceptions of how IPS impacted on the MDTs’ views of people with mental health difficulties. Findings suggest a shift in the ways in which MDTs view their clients. Initial fears about work capacity and risk shifted towards seeing people beyond the label of ‘service user’ and their diagnosis.

**Conclusions:**

It is contended that IPS is an approach that allows practitioners to engage with real recovery-practice and may be one key to unlocking how a recovery approach can truly trickle down and embed itself within mental health service provision and support mental health system reform.

## Introduction

The World Health Organization (WHO, 2021) outlines the bi-directional relationship between (un)employment and mental health, with employment identified as inherently linked to recovery. Employment can enhance a person’s mental health and quality of life by: enabling a structure to daily routine; contributing to a person’s identity and sense of self-worth, achievement and purpose; enabling autonomy; and building one’s social network (WHO, 2021). Despite the positive benefits of employment for mental health, people with mental health difficulties are consistently more likely to be unemployed due to a number of factors (Claussen et al., 1993; Organisation for Economic Co-operation and Development (OECD), 2015, 2021), including stigma and discrimination (Staiger et al., 2018), which in turn contributes to greater marginalization and a worsening of mental health for people with mental health difficulties (Virgolino et al., 2022; WHO, 2021).

The marginalization of people with mental health difficulties from the community has led to a call from the WHO and OECD to embed a more integrated, holistic, person-centred, recovery-oriented approach into the care of people with mental health difficulties, practices which respect the preference of people (OECD, 2015, 2021; WHO 2021). While there is an international call for the embedding of such practices within services (Coffey et al., 2019; Higgins et al., 2020; OECD, 2015, 2021; WHO 2021), it has been noted that the realization of recovery-oriented practices into routine practice is not straightforward, requiring systematic shifts in the current structures and ways of practice (Higgins et al., 2020). Indeed, it may require a fundamental paradigm shift in the conceptualisation and understanding of mental health difficulties, as well as, a rethinking on how to support people with mental health difficulties (Higgins & McGowan, 2014: p.66). Due to the nature of such a shift, it has been noted that there is a gap between the espoused policies and envisioning of recovery-oriented services and their actual implementation (Davidson, 2016).

One such recovery-related practice is Individual Placement and Support (IPS). IPS is a supported employment model for people with mental health difficulties. It is defined by eight core principles which include: a focus on the person’s strengths and preferences, no exclusion criteria to participation, rapid job search, benefits counselling, and ongoing support to the person and employers (Becker et al., 2019). The benefits of IPS have been validated by nearly three decades of international research (Bond et al., 2008, 2012; Brinchmann et al., 2020; Burns et al., 2007; Drake & Bond, 2011; Mueser et al., 2016; O’Day et al., 2017). A recent systematic review of 27 Randomised Controlled Trials of IPS evidences that IPS leads to higher rates of competitive employment, as opposed to volunteer work or sheltered employment, when compared to traditional vocational rehabilitation programs and is more effective than other employment approaches at getting people with severe and enduring mental health difficulties into work (Brinchmann et al., 2020). Such is the success of IPS that current studies have examined how to deliver IPS at scale (Bond et al., 2020) and its expansion into new and ever-broadening populations (Drake & Bond, 2017; Metcalfe & Drake, 2021), such as homeless people in the Housing First program (Poremski et al., 2017), people with post-traumatic stress disorder (Stroupe et al., 2021), and people with autism (McLaren et al., 2017).

While IPS is identified as both the most researched and also most successful supported employment model for people with mental health difficulties (Tighe & Murphy, 2020), a number of barriers remain in place to its successful implementation at both the systemic and service delivery levels (Bond et al., 2020; Bonfils, 2020). At the systemic level, national policy and legislation relating to employment may not support IPS, while there may also be a lack of infrastructure available to support the conjoined ways of working across employment, health, and other social services required by the program (Bond & Drake, 2014; Bond et al., 2020; Bonfils, 2020; Lockett et al., 2018). At the level of service delivery, the attitudes of clinicians about the IPS program, specifically their beliefs about the capability and capacity of people to work, can act as a barrier to IPS implementation if it leads them to resist the integration of the IPS Employment Specialist within the team (Bonfils, 2020; Priest & Lockett, 2020).

While these barriers have been examined internationally, existing literature touches only briefly and scantily on the way in which institutional logics are realized in practice or influence the implementation of IPS (for exceptions, see (Bonfils, 2020, 2021; Lockett et al., 2018). Institutional logics may be understood as “*the socially constructed, historical patterns of material practices, assumptions, values, beliefs, and rules by which individuals produce and reproduce their material subsistence, organize time and space, and provide meaning to their social reality”* (Thornton & Ocasio, 1999, p.804). Recently, Bonfils (2021) concluded that the institutional logics of mental health service practitioners became more recovery-oriented as they engaged more with the IPS program; however, they noted a lack of research and evidence around the extent to which engaging with IPS truly changes practice of those involved. Furthermore, questions remain about the detailed *how* and *why* of how such professional logics shifted. Current research does little to illustrate how these practices, values, and beliefs manifest and how IPS manages to gain a foothold within such settings. Questions abound about whether IPS is disruptive to institutions or whether it integrates itself into practice in other ways. This article aims to examine how IPS ESs and Occupational Therapy (OT) Managers managed to integrate and embed IPS within the Multi-Disciplinary Team (MDT) in the Irish mental health services.

## The current study context and background

Similar to international trends, recovery is the approach underpinning Irish mental health policies (Government of Ireland, 2006, 2020). In response to the high rates of unemployment within the Republic of Ireland among people with disability, including people with mental health difficulties (Central Statistics Office (CSO), 2016), Irish mental health policy identifies IPS as one way to support people with mental health difficulties with employment (Government of Ireland, 2006, 2020). IPS was introduced into Ireland through a pilot project titled *Integrating Employment and Mental Health Support* (Mental Health Reform, 2017). The project, which ran from 2015 to 2017 in four sites across Ireland, included 65 participants with mental health difficulties, with 33 of these participants obtaining at least one paid, competitive employment position throughout the duration of the project.

Subsequent to this, in 2018, IPS was scaled up as a national program, across the Irish mental health services through a funded reform process. This was part of a larger reform program, named the Service Reform Fund, a collaboration between: the Irish Department of Health; the Irish Department of Housing, Local Government and Heritage; the national Health Service Executive; Genio; and the Atlantic Philanthropies. The program aimed to integrate and embed more person-centred practices within the national mental health services, which included IPS, housing for people with mental health difficulties, and other recovery-related initiatives. As part of the program, IPS Employment Specialists were recruited from employment support organizations outside of the mental health services and their work was supervised by OT Managers within the mental health services, so that IPS could be integrated across the employment and health fields. With a view to enhancing the quality and efficacy of implementation of future developments in relation to IPS and other mental health recovery-focused initiatives, a research team were commissioned to examine the challenges and barriers, as well as the enablers and opportunities, to the implementation of IPS and the other recovery-related reforms. Supported by the [*Name of funder removed as identifiable. To be inserted after review]*, the study was to focus on generating insights into the implementation of the program as it was occurring.

This paper reports some aspects of the findings and focuses on the practical strategies that were taken by participants to integrate and embed IPS within the MDT. It also examines the nature of such integration and specifically explores whether IPS acted as a disruption or if it aligned with a well-established system, with well-established practices, values, and beliefs. The findings from other aspects of the research are currently being prepared for wider dissemination.

## Methodology

### Aim

The aim of this aspect of the study was to: (i) explore the context into which IPS landed in the mental health services; (ii) examine how IPS ESs and OT Managers navigated IPS implementation within this context; and (iii) explore the perspectives on the extent of change brought about through engagement in IPS.

## Study design

The findings that form the basis for this article arose from a larger study, the Service Reform Fund Action Research Study. The larger study, which itself was an integral part of a national reform program in the Republic of Ireland, was informed by an Action Research approach. Action Research may broadly be considered an approach to research that integrates theory and action, working with stakeholders directly to surface challenges and opportunities in an ongoing, cyclical way in order to address important social issues (Coghlan & Brydon-Miller, 2014). It was envisioned that such an approach would help bridge the ‘policy-implementation gap’ in which national policies and programs fail to get implemented due to issues at the local level (Grint, 2010; Hudson et al., 2019). This paper is guided by a qualitative, descriptive approach which is appropriate for research which aims to explore phenomenon while remaining close to the surface of the phenomenon (Bradshaw et al., 2017), grounding findings in the language of participants, using their own words to capture findings (Maxwell, 1992). This was particularly suitable to this study which examined IPS implementation issues as they were arising from the perspectives of key stakeholders.

## Data collection methods

Data for this article were collected through three qualitative, semi-structured focus groups (Rubin & Rubin, 2012), with a purposive sample involved with IPS. Such an approach was appropriate as it allowed the research team to ask questions related to IPS implementation, specifically experiences, challenges, barriers, opportunities, and enablers, while also allowing participants the opportunity to direct the conversations onto what they viewed as pertinent topics. The focus groups were guided by a topic schedule, developed by the research team (see Table 1) and conducted by two members of the research team (DS and NL). NL conducted one focus group with the IPS ESs. DS conducted the second focus group with the IPS ESs and a focus group with the OT Managers. Both interviewers were female. Both DS and NL hold PhDs in the social sciences, are employed as researchers, and have extensive experience in conducting qualitative research. Some of the participants may have known one or both of the researchers through their previous work in the area, although none had close relationships.

**Table 1:** List of topics and example questions included in the focus group schedule

Focus group topic	Example question(s)
Experiences in the role of OT Manager/Employment Specialist in relation to IPS	What have been your experiences with IPS so far?
Impact of IPS	What has been the impact of the IPS to date?
Local and national opportunities / enablers to IPS implementation	What is going well in terms of IPS? What are the opportunities of IPS?
Local and national challenges / barriers to IPS implementation	What are the challenges of IPS implementation?
Relationships of key stakeholders within the local mental health services to IPS	What level of traction and buy-in has IPS gained within the MDT?
Training and support needs	What are, if any, your training or support needs in relation to IPS?
Views on the sustainability and future of IPS	How do you view sustainability in terms of IPS?

## Sampling, recruitment, and participants

Purposive sampling was used to select the participants (Etikan et al., 2016). Any IPS ES in post in Ireland or OT Manager who line managed an IPS ES in Ireland as part of the reform program were eligible to participate in this study. It was envisioned that the purposive recruitment strategy would contribute to data saturation (Saunders et al., 2018). All IPS ESs and all OT Managers were informed about the study through e-mail either by the reform program manager or the OT Manager group representative, respectively. The email contained information about the study and an invitation to participate in the focus groups. Of the 24 IPS ESs in post at the time of data collection, 17 participated in two focus groups, which were organized to coincide with a community of practice event held at a hotel for this group. An additional focus group was conducted with 11 of 17 OT Managers, who supervised the IPS ESs at the time. This focus group was conducted as part of the OT Managers weekly group meeting in a health services’ building. It is unknown why the other seven IPS ESs and six OT Managers did not attend the event or participate in the focus group. The focus groups lasted between 53 and 75 min and were audio recorded. Field notes were made both during and after each focus groups. All focus groups represented a geographic spread across the Republic of Ireland, including both rural and urban areas.

### Data analysis

All focus groups were transcribed verbatim. Braun and Clarke’s (2006) six-step approach to inductive, semantic, thematic analysis informed the analysis of the data. This approach to analysis is data-driven, taking participants’ responses at face value, allowing the themes to emerge from the data (Braun & Clarke, 2006). First, the team immersed themselves in the data through reading and re-reading transcripts while note taking. From this process, initial codes were generated. These included descriptive codes describing chunks of the data, such as ‘beliefs about people with mental health difficulties’ and ‘providing information about IPS to the MDT’. The next step involved analyzing and organizing codes, combining them into coherent groups. Next, the team compared and contrasted codes and amalgamated them into themes. The next two steps involved reviewing the themes and defining and naming them. Through this, themes emerged, such as ‘“*There was a lot of, at the start, ‘Oh, sure they can’t work. They’re not able to. They’ll never be able to”*, describing the MDTs’ beliefs and assumptions around people and their capability to work, which is encapsulated in the findings section of this paper. QSR NVivo Version 11 (QSR International Pty Ltd., 2015) was used to store and manage the qualitative data.

## Ethics

All participants were informed of their rights as research participants, the purpose of the research and what participation involved, as well as the role of the researcher, both verbally by the researchers at the beginning of each focus group and through written informed consent forms. The researchers also explained about key issues related to voluntary participation, confidentiality, and data protection. Participants were encouraged to ask questions at any time related to the research study and their participation in it. All participants were provided with and signed informed consent forms. Ethical approval was granted from the Principal Investigator’s academic institution.

## Rigor

To contribute to the rigor of the qualitative analyses, all analyses were done by two members of the research team (DS and NL). In addition after data collection, the initial thematic findings were presented back to participants through member checking sessions (Cohen & Crabtree, 2006). These sessions provided participants the opportunity as a group to reflect on the findings, discuss them in a group setting, and to challenge or clarify any interpretations. In addition, in keeping with the qualitative research ethos, the sessions provided participants with the opportunity to consider how the findings related to their work in IPS implementation and whether any of the data could be used to help them work through challenges or capitalize on opportunities. The research is also reported in accordance with the Consolidated Criteria for Reporting Qualitative Research (COREQ) checklist (Tong et al., 2007).

## Results

Within this section, the results are presented within three themes. The first theme, titled ‘The context’, characterizes the context into which IPS landed. This was one marked by clinical resistance and skepticism towards IPS and clients’ capacity to work. The second theme examines the strategies and factors which were believed to facilitate the integration and embedding of IPS into the MDT and the Irish mental health services. The final theme, named ‘a recalibration of expectations’, elucidates participants’ perceptions of how IPS impacted on the MDTs’ views of people with mental health difficulties. Each participant is identified in the findings by a label (either ‘IPS ES’ or ‘OT Manager’) and a respondent code (R#). While some of the participants refer to people engaging with IPS as ‘service users’, we use the language of ‘client’ or ‘people with mental health difficulties’ to better mirror the language of IPS (Becker et al., 2019).

**The context: “*There was a lot of, at the start, ‘Oh, sure they can’t work. They’re not able to. They’ll never be able to.’”***.

This theme focuses on the context into which IPS was being implemented within the Irish mental health services. This was initially marked by a perceived lack of awareness and understanding around IPS, skepticism towards IPS and clients’ capacity to work, fears around its impact on client’s mental health and, at times, clinician-led decision making.

A lack of awareness and understanding within the MDT in terms of IPS was perceived by participants, with teams described as having “ *never heard of IPS*” (OT Manager R2) or:

*“None of them [on the MDT] have done any of the [IPS] training. So, none of them even know what fidelity is. They haven’t got an iota.” (IPS ES R2)*

Within this context, participants reported that the MDT initially had a number of concerns around IPS implementation. Questions abounded about the IPS ES’s role and competencies:

*“Just in terms of the teams, I would say resistance. Like when I met with the teams who were interested, the concern if you like, was about…‘This is all good on paper but at the end of the day, what is an Employment Specialist? Where do they train?’…I suppose that they were concerned about were boundaries…and just communication and…How are you going to ensure that?” (OT Manager R4)*

In addition, clinical confidentiality was a key issue within some of the MDTs being concerned about having a non-clinical person (the IPS ES) sitting within the MDT meeting. One IPS ES described how the MDT were “*a little bit wary of me”* as *“I didn’t have a clinical background”* (IPS ES R3).

In terms of the clients, participants described a lack of recognition or acknowledgement by the MDT of clients’ capacity to work. For instance, one OT Manager noted how “*competitive employment wouldn’t have necessarily been on the radar of a lot of our clinicians as being a realistic outcome”* (OT Manager R10). Another IPS ES described their experience with the MDT: *“There was a lot of, at the start, ‘Oh, sure they can’t work. They’re not able to. They’ll never be able to’”* (IPS ES R2).

Furthermore, there was a sense of fear of the potential risks associated with clients entering employment. Underscoring the MDTs’ fears was the assumption that once people became employed, they would become unwell, whether it be through relapse to addiction or a worsening of their mental health status due to the stress and pressure of working. In one case recounted by an ES, a person was not referred by the MDT to the IPS ES because there was the expressed assumption that if she became employed, she would relapse: *“‘Well, she’s a recovering alcoholic. She can’t work. She can’t be left on her own. You can’t leave her anywhere or she’ll be in the pub’*” (IPS ES R2). In the following vignette, another IPS ES recounts one person who was effectively prevented from work due to the medical consultant’s concerns about the person driving a car *if* they became unwell. In this case, the MDT’s concerns over-rode the client’s wishes to work:

*“Well, I had an experience with a client who wanted to drive and then I brought it to the MDT and the consultant didn’t want to let him drive…because of the risk he may pose if he was to become unwell…because the consultant holds clinical responsibility, you can understand it.” (IPS ES R9)*

In another story, the IPS ES, shared how the MDT had “*fear”* about the risk of a client having extra money and “*was going to spend it on very, very negative stuff”* (IPS ES R4).

The paternalistic, clinician-led decision making culture was not only evident in the above narratives but was also evident in the manner some referrals to IPS were made. Frequently, there appeared to be what one IPS Employment Specialist called “*a clash in opinion about what somebody thinks is best for a client and what the client wants for themselves”* (IPS ES R9). Two participants provided evidence of situations in which clinicians decided clients *should* work as opposed to the client themselves expressing such an interest: “*Often times we would get referrals from people who think a person should be working as opposed to the person actually themselves wanting to work”* (OT Manager R8). This led one ES to conclude: *“That’s something that needs to be hammered home in the Multi-Disciplinary Teams – That it has to be client-led, not clinically-led”* (IPS ES R6).

While there was a perceived fear of people becoming unwell due the pressure of returning to work, there was data to suggest that these fears were substantiated. In a small number of cases, the ESs did describe some clients who they believed experiencing a worsening of mental health difficulties and/or a relapse following returning to work. They emphasized that while “*that’s not everybody”*, “*it’s just to be aware that can happen”* (IPS ES R14). The IPS ESs were clear that part of their role involved supporting people to navigate challenges to their mental health and wellbeing throughout the employment process, with one noting how it is *“part of my job is also to help with that*” (IPS ES R2).

**Factors supporting the integration and embedding of IPS into the MDT: The need to come*“to terms with where their [MDT’s] mind-sets were”***.

In the face of the context described in theme one, a number of strategies and factors were identified that supported the integration and embedding of IPS into the MDT. These included strategies the IPS ESs and OT Managers used to navigate these clinical beliefs and practices, such as adopting an incremental approach, building trust and relationships, checking in with the MDT, reassuring the MDT about IPS ESs’ governance and competencies, and providing education and information to the MDT about IPS. It also included a number of factors that were believed to contribute to the acceptance of IPS by the MDT, including the personality and skills of the individual IPS ESs and the fact that IPS was a national program, with the accompanying accountabilities through IPS fidelity measures attached.

One key strategy used to embed IPS was the recognition that integrating was an ongoing process that required continued negotiation, achieved bit-by-bit versus all at once and one that, at times, required compromise. In most cases across the areas, it was also recognized that integration and embedding was a time intensive process, described as “*certainly very time consuming”* (OT Manager R1), and one which needed the allowance of time to unfold. There were accounts throughout the data of the ways in which IPS ESs built relationships and engaged collaboratively with the MDT in order to generate trust and goodwill towards IPS. There was an expressed need to come “*to terms with where their [MDT’s] mind-sets were”* (IPS ES R2). In one case, the IPS ES described how the MDT was “*very aware”* that they did not have a clinical background and that they attempted to generate buy-in amongst the MDT through *“building trust”* with the various heads of departments, nurses, social workers, and OTs.

*“So, I suppose we did the way where we went and we met the heads of departments, we talked to them, more or less asked for permission to talk to the nurses, the social workers, the OTs. We did it that way and as time went by, people start to recognize you more in the corridor, they’ll start to approach you…So, it was about building the trust and it’s taken a bit of time, but I mean I’ve a full caseload, my waiting list is on hold. So, obviously, you know, you have to be positive.” (IPS ES R3)*

One OT Manager described having to “*compromise a bit*” to address concerns amongst the MDT around governance which included limiting IPS “*access to the file”* and only allowing them to participate in “*part of the [MDT] meeting”* (OT Manager R6). Such a compromise meant that IPS could not be implemented fully in terms of IPS fidelity criteria which indicates that the IPS ES should be an embedded and participatory member of the MDT. In this case, the OT Manager was viewing the integration process in a step-wise fashion, with the aim that full fidelity would occur in an incremental manner. Another OT Manager described their strategy of perpetually checking in with the MDT as “*repeating the core points…and constantly sort of addressing the concerns as they were raised and being willing to respond quickly to those concerns and proactively sort of then checking in once staff had commenced*” (OT Manager R1). Such an approach suggests that relationship building with the MDT was a continuing, evolving process. In building relationships and engaging with the MDT, the IPS ES’s unique personality and qualities also proved to be an important factor, with one OT Manager how the IPS ES’s “*personality and skills and all, you know, when embedding into the team really helped it*” (OT Manager R6).

Another strategy for facilitating the integration of the IPS ES involved reassuring the MDT about the IPS ES’s role, the quality of governance, and their competencies. Such a strategy assuaged the MDT’s concerns in some areas. In one case, the presence of the OT Manager on the interview board for the IPS ES was considered reassuring (OT Manager R4). Another OT Manager described how having a clear management structure for the IPS ES was “*a big buy-in for the staff on the ground…they know that the person is being supervised*” and that “*the line management structure is really, really clear”* (OT Manager R8).

In the context of a perceived lack of understanding around IPS, OT Managers and IPS ESs provided information about the program to IPS. Participants recounted how they did presentations to the MDT and “*put together a kind of package and sent it out to all the teams and asked…if they wanted an IPS worker”* (OT Manager R9). There were training gaps identified in terms of IPS, with a consensus that there needed to be accessible training for the MDT: “*I would like for the Multi-Disciplinary Team maybe to do some more training around IPS just to get an understanding of what it is”* (IPS ES R6). It was perceived that OT Managers and IPS ESs spent a lot of time “*repeating of work”* (OT Manager R9), as there was no coordinated national information available regarding IPS. It was proposed that generating buy-in for IPS could be simplified “*just in terms of selling to the team what IPS is”* (OT Manager R2) if there was a national information package that could be distributed to MDTs. One participant described how such a package would encompass, *“The evidence. ‘This is the references. This is what IPS is’”* (OT Manager R2). The provision of such education and information was seen to be a supporting factor in the acceptance of IPS ES into the MDT. While it was believed that there needed to be accessible training offered regularly for the MDT, it was contended by some participants that in-person training was not necessarily required for the entire team, and that “*a good information package…may be adequate*” (OT Manager R11) or “*a standardized piece…might be more the way forward*” (OT Manager R4).

The fact that IPS was a national project was a further factor that was believed to contribute to the acceptance of IPS by the MDT, described as “*reassuring for the team*” in at least one area, meaning there was a “*national data…confidentiality agreement, a data sharing agreement”* (OT Manager R4). Yet another participant described how the “*fidelity scale was very useful…in some of the education*” to MDTs (OT Manager R2). These quotes give a sense that the power of IPS in being both a national program, with attached fidelity, was a factor that contributed to buy-in from the MDT.

**A recalibration of expectations: “*Their [MDT] eyes are opened as well and like they weren’t aware actually at how capable people are****”*.

Once IPS ESs and OT Managers managed to create an opportunity for IPS to unfold within the mental health services, many participants reported a perceived change in perspectives within the MDT in terms of how they viewed people using mental health services and employment. Such changes were described as shedding a light on unconscious biases held by the MDT, challenging their assumptions, and enabling members of the MDT to see clients beyond the mental health diagnosis and ‘service user’ label.

Narratives suggested that the assumptions around people and employment (detailed in the first section of the findings) were challenged, leading to “*surprise”* within some of the teams. This is exemplified in the following comment .

*“I think the team have been surprised and very interested and interested in what kinds of jobs and surprised at the skills that they didn’t know that [the people they support] had…” (OT Manager R2)*

One IPS ES noted that what the MDT in their area, *“are saying that they just weren’t aware that the [person’s] capability was actually there”* (IPS ES R16). In another case, the IPS ES described how the MDT’s “*eyes are opened as well”*:

*“What I’ve noticed is that the MDT members are actually quite, their eyes are opened as well and like they weren’t aware actually at how capable people are and that was the biggest thing for me…” (IPS ES R16)*

Interestingly, this IPS ES reflects on being “*not quite sure what’s happening but…it wasn’t there before [IPS]”* (IPS ES R16).

This surprise led to a perceived shift in expectations from the MDT about people with mental health difficulties, summarized by one OT Manager as *“a recalibration from the team about their own expectations about what the [person they support] is capable of”* (OT Manager R1). There was a sense within some narratives that IPS enabled the MDT to see people beyond the ‘service user’ label, deficits, and diagnosis to viewing people with mental health difficulties as “*people just like us who can work”* (OT Manager R2).

*“So, it’s just really practical things and I think maybe it’s making the team...despite all this talk of recovery, I think it’s making the team actually realize that these are people just like us who can work. So, I think, you know, from a real basic level that it’s letting the skills of the person shine.” (OT Manager R2)*

These quotes suggest that through engaging with IPS, ingrained beliefs and unconscious biases amongst the MDT about people and their capacity were both uncovered and challenged. In the opinion of the interview participants, the IPS process helped “*really clarify bias…in terms of how we look at [the people we support]”*, giving respect and primacy to clients’ personal preferences for work or not (OT Manager R8). IPS was portrayed by some participants as a mechanism for grounding the recovery ethos, allowing the MDT to see clients more holistically, as described by this OT Manager:

*“The impact on the team…it was amazing…I think that impact is really important in terms of in recovery and development recovery services. Like sometimes you see these teams and ‘Oh, they’re not recovery’, ‘They’re not this’ and ‘No, let’s give them more training to be recovery’. Like actually, you know, having a role and getting on with it, once they see it happening, they buy in...” (OT Manager R4)*

This breaking down of the barrier between ‘service user’ and ‘clinician’ led one IPS ES to conclude that the MDT “*have a better relationship with the clients now”* because “*there’s more of an understanding, it isn’t ‘them’ and ‘us’…it’s…we’re all people”* (IPS ES R4).

As these narratives evidence, the success story of clients with IPS was perceived to shift resistance amongst the MDT across the areas: “*That [positive experience of clients] sells it then straight away”* (OT Manager R10). In one quote, the IPS ES describes how the MDT have “*embraced”* IPS, although it is “*a totally different discipline [area of focus] for them”*; however, they seem to credit this to a reduction in the role of medication and other therapeutic intervention as a result of IPS.

*“So, with that I really feel, I suppose very positively about how the MDT has embraced it, like, it’s a totally different discipline for them because most of the time they’re talking about medical trauma and whatever but, you know, I’ve had people in my audit quote that they have individuals have reduced medication and reduced their time, their amount of therapeutic interventions as a result of IPS….” (IPS ES R12)*

The positive impact of these changes was reflected in a large number of referrals and uptake in IPS across their regions, which then contributed to waiting lists in a number of areas. It also led to a call from other MDTs for IPS ESs within their clinical team, which also resulted in there not being enough IPS ESs available to match the demand.

*“We’ve moved from the space where Community Teams were asking, ‘What is IPS?’ and ‘Why do we need it?’ to ‘Why haven’t we got an Employment Specialist?’” (OT Manager R1)*

## Discussion

It is reasonable to assume that no matter how strong the evidence base for IPS is, the model cannot simply be dropped into existing mental health services and expected to take hold, without reference to institutional logics that shape the identities, beliefs, values and practice of professionals (Bonfils, 2021). Few studies, however, provide empirical data to illustrate how people tasked with implementation IPS address institutional logics or the extent to which engaging with IPS may influence and change professionals’ practice (Bonfils, 2021). This study details the institutional context for IPS implementation in the Republic of Ireland and the strategies implementers used to navigate this context. Institutional theory predicts that as patterns of work or other institutionalized activities become established and taken-for-granted, they become highly resistant to change (Zucker, 1987). This was certainly the case in this study as findings indicate that the context was marked by clinical resistance and wariness towards IPS and, similar to the findings of Bonfils (2020), some members of the MDT screened potential IPS clients, pre-emptively deciding who they believed was or was not ready for employment.

These findings not only demonstrate an overwhelming sense of doubt among the MDT about clients’ capacity for work, but they are also not in keeping with the espoused Irish national recovery and rights-based policy that highlight the importance of social inclusion (Government of Ireland, 2006, 2020; O’Feich et al., 2019). The fear that entering employment would negatively impact the person’s mental health and put their recovery at risk also bolster other research within Ireland in which Tighe & Murphy (2020) could not identify a single member of the MDT who believed that people with mental health difficulties should go directly into paid employment, instead preferring a step-wise entrance through work experience or further training. Such beliefs are in contradiction to the IPS ‘ready to work’ tenet that any client who has the willingness can and should be facilitated to engage with the IPS process (Becker et al., 2019).

These views and practices may reflect what Cohen & Cohen (1984) refer to as ‘the clinicians’ illusion’, where clinicians as a result of frequently seeing people at the most acute stages of their mental distress, do not believe that people are able to work or that work can play a crucial role in their recovery journey. They may also be a result of clinicians viewing recovery through the medical lens as the absence of the clinical symptoms. Such professional narratives of inability and pessimism in relation to people with mental health difficulties contrasts starkly to a holistic understanding recovery, in terms of a focus on strengths, possibility, and hope (Ellison et al., 2018; Watts & Higgins, 2016) and the value of independent living, employment, and social life to a person (Kern et al., 2009), which characterized the OT Managers’ and IPS Employment Specialists’ approach.

In addition to this, IPS fidelity mandates the integration of the IPS ES within the Mental Health Team. Similar to other studies, there was a resistance and reluctance amongst the MDTs around working with and integrating the IPS ES, a non-clinical role, into the team (Bond et al., 2020; Priest & Lockett, 2020; Shepherd et al., 2012). This led to compromises being made in some areas, a hindrance to IPS fidelity. Similar findings were reported by Bonfils (2020) who found that Mental Health Teams in their study were skeptical of IPS ESs, seeing them as “*outsiders*” and even seeing IPS as a “*waste”* of resources, making integration challenging or even impossible (Bonfils, 2020). Such beliefs not only act as a barrier to the successful embedding of IPS locally, but can lead to difficulties in scaling it beyond a local region (Boardman & Rinaldi, 2013; Bond et al., 2020; Shepherd et al., 2012).

Tackling such ingrained beliefs and practices is not a minor challenge, as they go right into the core of some professionals’ practice. In many ways, it would be fair to say that IPS requires a seismic shift across mental health and employment services, including changes in clinicians’ beliefs about clients and their capacity and capabilities (Boardman & Rinaldi, 2013). While the medical model and recovery ethos are not in complete opposition, as diagnosis and medication may be a part of some people’s recovery journey, a medical model approach has been identified internationally as a significant barrier to successful IPS implementation, making it critical to understand how clinicians who do have such a mind-set can be engaged with throughout the IPS program (Boardman et al., 2003; Boardman & Rinaldi, 2013; Bonfils, 2020, 2021; Bonfils et al., 2017; Boyce et al., 2008; Craig et al., 2014; Knaeps et al., 2012; Marwaha et al., 2009; Menear et al., 2011; Modini et al., 2016; Moen et al., 2020; O’Brien et al., 2003; Rinaldi et al., 2008, 2010; Shepherd et al., 2012; Tighe & Murphy, 2020; van Erp et al., 2007).

Findings in this study highlighted a number of strategies and factors which helped with the gradual introduction of IPS within the MDT. Factors like the national remit of the IPS program and its attached accountabilities through IPS fidelity measures were perceived to play a role in its acceptance. IPS ESs and OT Managers also relied on a number of strategies to garner buy-in, namely providing education and information, reassuring the MDT about the line management and competencies of the IPS ESs, and building trust in relationships in order to gain an initial foothold within services. We contend that by underscoring the nature of its evidence-base and by providing reassurance around governance, IPS was aligned with the MDT’s beliefs and practices, as opposed to challenging them. However, once the IPS program was underway, the success of clients engaged with the IPS work challenged clinical assumptions and led to a ‘recalibration of expectations’ about the capacity and capabilities of people with mental health difficulties. In so doing, a sense of a shared humanity emerged, with reports that the MDT were now seeing beyond the diagnosis and label of ‘service user’. These findings support, that when IPS is done well, it can help break down this ‘clinicians’ illusion’ and contribute to more positive attitudes about clients’ capacity and capability for work by providing clinicians with the opportunity to both observe and participate in the vocational aspects of a client’s recovery (Gladman et al., 2015). The findings also lend backing to the importance of providing support and training at the outset of IPS implementation, partly in order to assist clinicians in understanding their role in implementation (Johnson-Kwochka et al., 2017).

While some changes within the MDT were evident, questions do arise about the level of change and the extent to which engagement with the program infiltrates and changes core practice (Bonfils, 2021). In other words whether the change reported constitutes a first order change characterized by a superficiality and a high susceptibility to reverting back to the previous ways of doing things or a second order change, characterized by a true, radical, and genuine change of values and practice (Argyris, 1996). Although we suggest that IPS fits in with and conformed to some aspects of the MDTs’ institutional logics, we also contend that its ability to do so only allowed it to gain an initial foothold into services, allowing the MDT to engage with IPS and see clients’ succeed. The concept of ‘small wins’ offers a lens to understand this process and how they have the potential to make big changes in terms of institutional logics. While such individual wins may seem ‘small’ and ‘unimportant’, authors have contended that they have the potential to snowball by accumulating, scaling up, broadening or deepening (Termeer & Dewulf, 2019).

We propose that after gaining an initial foothold and through engagement with IPS, practices within the MDT truly did change to the second order level (Argyris, 1996) and mind-sets shifted from fear and risk of clients getting into employment to one of recognizing clients’ capability and abilities. The shift in practitioners’ mind-sets evidenced in this study may also speak to what Kania et al., (2018) term ‘mental models’ or ways of thinking about the world. While other levels such as policy, practice, and resource flow, including relationships and power dynamics are challenging to change because of the their inter-related nature, shifting mental models is described as the most crucial level to shift if systemic reform is to truly take hold and embed (Cabaj, 2019; Kania et al., 2018). This shift from viewing of people with mental health difficulties as incapable to capable is also evidenced in Bonfils’ (2020) study in Denmark in which the author described mental health managers’ views moving more in line with IPS principles, with one mental health manager in that study voicing a markedly similar comment to a participant in the current study: “*We notice that patients can do more than we sometimes think”* (Bonfils, 2020, p.10).

The participants in our study voiced moving vignettes of clients’ transformation and described the way these conflicted with the MDTs’ previously-held beliefs and expectations about clients. In so doing, IPS appeared to ground the recovery ethos in an accessible way and provided members of the MDT with real-life examples of success stories that helped challenge and shift attitudes and practices towards a more recovery ethos. Indeed, authors have highlighted the importance of moving beyond recovery theory and providing practitioners with the opportunity to engage in a meaningful way with recovery-oriented practices in order to enhance their self-efficacy and ability to change behavior (Higgins et al., 2020).

While IPS may be one key to unlocking how a recovery approach can truly trickle down and embed itself within mental health service provision supporting mental health reform, when we dig deeper into what actually ‘convinced’ the MDT to engage with IPS, the story becomes less clear. There was a sense from *some* narratives that the merits of IPS lie in terms of symptom reduction and employment as a complimentary ‘therapy’. This is also supported by Bonfils (2020; 2021) studies in Denmark which note that although the mind-sets of some mental health managers shifted, there was an overall sense that employment continued to be seen as a *“parallel service to treatment”*, as something supplementary to the real work of the mental health services which centred on their official remit of the treatment and care of mental health difficulties (Bonfils, 2020, p.9). Within that study, the authors concluded that IPS then had to *“fit in”* to the existing mental health services (Bonfils, 2020, p.1). While the medical model has been viewed as a barrier to implementation, this suggests that IPS, in fact, aligns with the MDT’s institutional logic, making it far more comfortable with the medical model than disruptive to it. This begs the question about whether this was the true reason why IPS may be readily absorbed by the MDT? This question requires future research to uncover issues of alignment versus disruption.

## Limitations

This study relies on a qualitative, descriptive approach which has some inherent limitations. In many instances, participants, in addition to recounting their own direct experiences, recounted their views and inferences about the perceived feelings, attitudes, and behaviors of the MDT. There is unavoidably subjectivity in these accounts, as all findings were analyzed through the lenses of the IPS ESs and OT Managers. This study was limited in that clinicians sitting on the MDT were not interviewed; thus, their views on the topic could not be explored. In addition, IPS clients were not interviewed; therefore, the research does not explore if, and if so, how, IPS clients may have experienced any changes in support or attitudes from clinicians related to their participation in IPS. This is a worthwhile area for future research. By interviewing the majority of IPS ESs and OT Managers across differing geographical locations, there was a degree of triangulation of data whereby accounts made by one were frequently supported by accounts from others; thus, this study may be considered a rigorous account of the views of the IPS ESs and OT Managers.

## Conclusions

In this article, we have explored how IPS ESs and OT Managers navigated the integration and embedding of recovery-oriented IPS in traditionally recovery-oriented MDTs. Within this study, a preconception prevailed amongst the MDT that people with mental health difficulties were not ready or able to work (that they would fail) or that employment would cause a person to become unwell. Concerns were also present about the role of the IPS ES, especially in relation to the MDT. We have specifically examined what factors and strategies enabled the integration and embedding of IPS into the MDT. We contend that the evidence-based nature of IPS and the strategies used by IPS ESs and OT Managers reassured the MDT that IPS was non-threatening to clinicians’ core identities. We recounted stories of IPS clients’ successes and the ways in which these surprised and challenged the MDT. We have posited that this allowed for the success of ‘small wins’ to drive the program forward. While there was evidence that IPS was transforming clinical beliefs in regard to people with mental health difficulties, there was also data to suggest that IPS was actually simply aligning to the MDT’s institutional logic in terms its success with ‘symptom reduction’. Therefore, this article raises questions about the extent to which IPS transforms or simply adheres to medical model logic. Few studies have actually explored the *why* and *how* IPS manages to gain a foothold into the MDT and what happens when it does. This study has gone in some way to addressing this gap by providing empirical data, triangulated across a number of data sources. Furthermore, the findings may be applied in various social systems and contexts to understand how a ‘small wins’ approach may incrementally tackle big challenges.

## Electronic supplementary material

Below is the link to the electronic supplementary material.

Supplementary Material 1

Supplementary Material 2

## Data Availability

It is not possible to make the raw data from this study publicly available for the following reasons. Given the relatively small size of the population and sample, making complete focus groups transcripts publicly available could compromise confidentiality and/or participant privacy. Furthermore, the granting of ethical approval for this study did not include permission to publicly share the raw data.
