# Supplementary material for: “These are people just like us who can work”: Overcoming clinical resistance and shifting views in the implementation of Individual Placement and Support (IPS)
Source: Adm Policy Ment Health. 2022 Jul 5;49(5):848–60. doi: 10.1007/s10488-022-01204-3 (PMC9393135; doi:10.1007/s10488-022-01204-3)
Supplement: Supplementary file 1 — Supplementary Material 1 [file 10488_2022_1204_MOESM1_ESM.doc]

| **Topic, Item No., and Guide Questions/Description** | **Reported in Manuscript** |
| --- | --- |
| **Domain 1: Research team and reflexivity**  *Personal Characteristics* | |
| 1. Interviewer/facilitator: Which author/s conducted the interview or focus group? | P.6. “The focus groups were guided by a topic schedule, developed by the research team (see Table 1), and conducted by two members of the research team (DS and NL). NL conducted one focus group with the IPS ES. DS conducted the second focus group with the IPS ES and a focus group with the OT Managers.” |
| 2. Credentials: What were the researcher’s credentials? E.g. PhD, MD | P.6 “Both DS and NL hold PhDs in the social sciences, are employed as researchers, and have extensive experience in conducting qualitative research |
| 3. Occupation: What was their occupation at the time of the study? | P.6 “both DS and NL hold PhDs in the social sciences and have extensive experience in conducting qualitative research.” |
| 4. Gender: Was the researcher male or female? | P.6 “Both interviewers were female” |
| 5. Experience and training: What experience or training did the researcher have? | P.6 “Both DS and NL hold PhDs in the social sciences, are employed as researchers, and have extensive experience in conducting qualitative research |
| *Relationship with participants* | |
| 6. Relationship established: Was a relationship established prior to study commencement? | P.6 “Some of the participants may have known one or both of the researchers through their previous work in the area, although none had close relationships.” |
| 7. Participant knowledge of the interviewer: What did the participants know about the researcher? e.g. personal goals, reasons for doing the research | P.6 “Some of the participants may have known one or both of the researchers through their previous work in the area, although none had close relationships.” |
| 8. Interviewer characteristics: What characteristics were reported about the interviewer/facilitator? e.g. Bias, assumptions, reasons and interests in the research topic | p.8 “All participants were informed of their rights as research participants, the purpose of the research and what participation involved, as well as the role of the researcher, both verbally by the researchers at the beginning of each focus group and through written informed consent forms.” |
| **Domain 2: study design**  *Theoretical framework* | |
| 9. Methodological orientation and Theory: What methodological orientation was stated to underpin the study? e.g. grounded theory, discourse analysis, ethnography, phenomenology, content analysis | P.5 “The larger study, which itself was an integral part of a national reform programme in the Republic of Ireland, was informed by an Action Research approach.” |
| *Participant selection* | |
| 10. Sampling: How were participants selected? e.g. purposive, convenience, consecutive, snowball | P.7 “Purposive sampling was used to select the participants (Etikan et al., 2016).” |
| 11. Method of approach: How were participants approached? e.g. face-to-face, telephone, mail, email | P.7 “All IPS ESs and all OT Managers were informed about the study through e-mail either by the reform programme manager or the OT Manager Group representative, respectively.” |
| 12. Sample size: How many participants were in the study? | P.7 “Of the 24 IPS ESs in post at the time of data collection, 17 participated in two focus groups, which were organised to coincide with a community of practice event held at a hotel for this group. An additional focus group was conducted with 11 of 17 OT Managers, who were the supervisors of the IPS ESs at the time.” |
| 13. Non-participation: How many people refused to participate or dropped out? Reasons? | P.8 “It is unknown why the other seven IPS ESs and six OT Managers did not attend the event or participate in the focus group.” |
| *Setting* | |
| 14. Setting of data collection: Where was the data collected? e.g. home, clinic, workplace | P.7-8. A hotel and a health services’ building |
| 15. Presence of non-participants: Was anyone else present besides the participants and researchers? | No, no one else was present besides the participants and researchers. |
| 16. Description of sample: What are the important characteristics of the sample? e.g. demographic data, date | P.7-8 All demographic information available about the participants is reported. |
| *Data collection* | |
| 17. Interview guide: Were questions, prompts, guides provided by the authors? Was it pilot tested? | P.6 “The focus groups were guided by a topic schedule, developed by the research team (see Table 1)…” |
| 18. Repeat interviews: Were repeat interviews carried out? If yes, how many? | No, repeat interviews were carried out. |
| 19. Audio/visual recording: Did the research use audio or visual recording to collect the data? | P.8 “The focus groups lasted between 53 minutes and 75 minutes and were audio recorded.” |
| 20. Field notes: Were field notes made during and/or after the interview or focus group? | P.8 “Field notes were made both during and after each focus groups.” |
| 21. Duration: What was the duration of the interviews or focus group? | P.8 “The focus groups lasted between 53 minutes and 75 minutes and were audio recorded.” |
| 22. Data saturation: Was data saturation discussed? | P.7 “It was envisioned that the purposive recruitment strategy would contribute to data saturation (Saunders et al., 2018).” |
| 23. Transcripts returned: Were transcripts returned to participants for comment and/or correction? | No, the transcriptions were not returned to participants but all initial findings were shared for comment and clarification via member checking sessions (p.9). |
| **Domain 3: analysis and findings**  *Data analysis* | |
| 24. Number of data coders: How many data coders coded the data? | P.9 “To contribute to the rigor of the qualitative analyses, all analyses were done by two members of the research team (DS and NL).” |
| 25. Description of the coding tree: Did authors provide a description of the coding tree? | P.8 Yes. |
| 26. Derivation of themes: Were themes identified in advance or derived from the data? | P.8 “All focus groups were transcribed verbatim. Braun and Clarke’s (2006) six-step approach to inductive, semantic, thematic analysis informed the analysis of the data. This approach to analysis is data-driven, taking participants’ responses at face value, allowing the themes to emerge from the data (Braun & Clarke, 2006).” |
| 27. Software: What software, if applicable, was used to manage the data? | P.8 “QSR NVivo Version 11 (QSR International Pty Ltd., 2015) was used to store and manage the qualitative data.” |
| 28. Participant checking: Did participants provide feedback on the findings? | Yes, p.8. “In addition after data collection, the initial thematic findings were presented back to participants through member checking sessions (Cohen & Crabtree, 2006)...” |
| *Reporting* | |
| 29. Quotations presented: Were participant quotations presented to illustrate the themes / findings? Was each quotation identified? e.g. participant number | Yes, throughout the findings.  P.9 “Each participant is identified in the findings by a label (either ‘IPS ES’ or ‘OT Manager’) and a respondent code (R#).” |
| 30. Data and findings consistent: Was there consistency between the data presented and the findings? | Yes. |
| 31. Clarity of major themes: Were major themes clearly presented in the findings? | Yes. |
| 32. Clarity of minor themes: Is there a description of diverse cases or discussion of minor themes? | Yes. |
