# Supplementary material for: “These are people just like us who can work”: Overcoming clinical resistance and shifting views in the implementation of Individual Placement and Support (IPS)
Source: Adm Policy Ment Health. 2022 Jul 5;49(5):848–60. doi: 10.1007/s10488-022-01204-3 (PMC9393135; doi:10.1007/s10488-022-01204-3)
Supplement: Supplementary file 2 — Supplementary Material 2 [file 10488_2022_1204_MOESM2_ESM.docx]

April 2022

**Re: Manuscript ID #APMH-D-22-00024**

Dear Dr. Bond,

Thank you and the reviewers for reviewing our manuscript entitled ‘*“These are people just like us who can work”*: Overcoming clinical resistance and shifting views in the implementation of Individual Placement and Support (IPS)’*.* We appreciate your feedback and have responded to each comment as detailed in the table on the following pages and marked in red in the resubmitted manuscript. We hope this revised resubmission addresses your comments. Please contact me with any further comments or queries.

Sincerely,

The Authors
